# Facile synthesis of g-C_3_N_4(0.94)_/CeO_2(0.05)_/Fe_3_O_4(0.01)_ nanosheets for DFT supported visible photocatalysis of 2-Chlorophenol

**DOI:** 10.1038/s41598-019-46544-7

**Published:** 2019-07-15

**Authors:** Jamshaid Rashid, Nadia Parveen, Aneela Iqbal, Saif Ullah Awan, Naseem Iqbal, Shamraiz Hussain Talib, Naveed Hussain, Bilal Akram, Ata Ulhaq, Bilal Ahmed, Ming Xu

**Affiliations:** 1 Department of Environmental Science, Faculty of Biological Sciences, Quaid-i-Azam University, Islamabad, 45320 Pakistan; 2 Key Laboratory of Geospatial Technology for the Middle and Lower Yellow River Regions, College of Environment and Planning, Henan University, Keifeng, 475004 China; 3 Department of Electrical Engineering, NUST College of Electrical and Mechanical Engineering, National University of Science and Technology (NUST), Islamabad, 54000 Pakistan; 4 US-Pakistan Centre for Advanced Studies in Energy (USPCAS-E), National University of Sciences and Technology, Islamabad, Pakistan; 5 Department of Chemistry, Tsinghua University, Beijing, 100084 P.R. China; 6 State Key Laboratory of New Ceramics and Fine Processing, School of Material Science and Engineering, Tsinghua University, Beijing, P.R. China; 7 Department of Physics, Lahore University of Management Sciences (LUMS), Lahore, 54792 Pakistan

**Keywords:** Pollution remediation, Photocatalysis, Two-dimensional materials

## Abstract

Visible light active g-**C**_**3**_**N**_**4(0.94)**_**/CeO**_**2(0.05)**_**/Fe**_**3**_**O**_**4(0.01)**_ ternary composite nanosheets were fabricated by facile co-precipitation routes. The density functional theory (DFT) computations investigated changes in geometry and electronic character of g-C_3_N_4_ with CeO_2_ and Fe_3_O_4_ addition. Chemical and surface characterizations were explored with XRD, XPS, SEM, TEM, PL, DRS and Raman measurements. DRS and PL spectroscopy evidenced the energy band gap tailoring from 2.68 eV for bulk g-C_3_N_4_ and 2.92 eV for CeO_2_ to 2.45 eV for the ternary nanocomposite. Efficient electron/hole pair separation, increase in red-ox species and high exploitation of solar spectrum due to band gap tailoring lead to higher degradation efficiency of g-**C**_**3**_**N**_**4(0.94)**_**/CeO**_**2(0.05)**_**/Fe**_**3**_**O**_**4(0.01)**_. Superior sun light photocatalytic breakdown of 2-Chlorophenol was observed with g-C_3_N_4_ having CeO_2_ loading up to 5 wt%. In case of ternary nanocomposites deposition of 1 wt% Fe_3_O_4_ over g-C_3_N_4_/CeO_2_ binary composite not only showed increment in visible light catalysis as predicted by the DFT studies, but also facilitated magnetic recovery. The g-**C**_**3**_**N**_**4(0.94)**_**/CeO**_**2(0.05)**_**/Fe**_**3**_**O**_**4(0.01)**_ nanosheets showed complete mineralization of 25 mg.L^−1^ 2-CP_(aq)_ within 180 min exposure to visible portion of sun light and retained its high activity for 3 consecutive reuse cycles. The free radical scavenging showed superoxide ions and holes played a significant role compared to hydroxyl free radicals while chromatographic studies helped establish the 2-CP degradation mechanism. The kinetics investigations revealed 2.55 and 4.04 times increased rate of reactions compared to pristine Fe_3_O_4_ and CeO_2_, showing highest rate constant value of 18.2 × 10^−3^ min^−1^ for the ternary nanocomposite. We present very persuasive results that can be beneficial for exploration of further potential of **g-C**_**3**_**N**_**4(0.94)**_**/CeO**_**2(0.05)**_**/Fe**_**3**_**O**_**4(0.01)**_ in advance wastewater treatment systems.

## Introduction

Energy, changing climate and water purification are becoming worldwide challenges to fulfill the demands of ever growing societies. In past few years water pollution has become an imperative problem for the environmental scientists across the globe. Wastewater is an essential by-product of modern industry and plays an important role as a pollution source in the environment. For instance, 2-Chlorophenol (2-CP) is an ubiquitous pollutant due to its widespread release into the environment as a by-product during the manufacturing of plastics, dyes, pulp and paper industry as well as petroleum refining^1,2^. 2-CP possessing toxic natural properties, badly affects the biotic life forms along the food chain^3–6^. Moreover, it is difficult to remove 2-CP using conventional treatment techniques because of their sensitivity to environmental factors, slow mode of action, high budgetary requirements and/or production of unwanted solid residues^7–9^. Among the diverse sustainable developments of recent years, semiconductor photocatalysis for harnessing the virtually endless solar power resource has emerged as a technology with immense potential for power generation and environmental cleanup^10^. Photocatalysis in particular, due to the non-selective behaviour toward organic contaminants is investigated as the most favourable technology for destructive removal of phenols and phenolic compounds from wastewater^11,12^. For quite some years, the choice ultra violet (UV) and visible light (VL) active photocatalysts comprised of variants of semiconductors like TiO_2_, ZnS, Fe_2_O_3_, CdS, Bi_2_WO_6_, InVO_4_, Ta_3_N_5_, TaON^13,14^.

While searching for vigorous and VL dynamic semiconductor photocatalysts g-C_3_N_4_, has generated impression of enthusiasm among scientific societies as the next-generation photocatalyst, owing to its high physicochemical stability, attractive optoelectronic properties, and tunable niche^15–27^. The semiconductor catalyst can be synthesized by single step temperature controlled polymerization of low cost and readily available nitrogen rich precursors^28–31^. Thus the surface chemistry of g-C_3_N_4_ could be manipulated with ease through molecular level engineered surface designs. Furthermore, g-C_3_N_4_ bears the lowest energy band gap among its seven polymeric phases, owing to sp^2^-hybridized C and N having π-conjugated electronic systems. Compared to TiO_2_, g-C_3_N_4_ band gap is considerably small i.e., 2.7–2.8 eV, responsible for absorption in 450–460 nm ranges of visible spectrum^32^. Unfortunately pristine g-C_3_N_4_ suffers from some limitations which hinder the wide scale use of g-C_3_N_4_ involving slow efficiency of solar light utilization (>460 nm) and high electron/hole pair’s recombination following photo-excitation (in picoseconds). Furthermore, separation of non-magnetic photocatalyst from huge volumes of treated solutions also halts its practical implications at larger scale^33,34^. The degradation potential of g-C_3_N_4_ can also be enhanced significantly by pairing up with a variety of semiconductors like Fe_3_O_4_, TiO_2_, AgI, InVO_4_ and WO_3_ due to efficient electron hole pair separation across the heterojunction between the semiconductors^35–38^. Moreover, coupling with Fe_3_O_4_ is explored owing to its stability, cost effectiveness and facile recovery of the resulting photocatalyst from the treated solution and absence of chemical and energy intensive post recovery activation procedures^39,40^. Also recently, the simultaneous coupling of two kinds of semiconductors into g-C_3_N_4_ has attracted considerable interest^41–45^. To our literature survey, this is the first report on fabrication of g-**C**_**3**_**N**_**4(0.94)**_**/CeO**_**2(0.05)**_**/Fe**_**3**_**O**_**4(0.01)**_ for applications in wastewater treatment yet. Hence, this investigation reports a novel g-**C**_**3**_**N**_**4(0.94)**_**/CeO**_**2(0.05)**_**/Fe**_**3**_**O**_**4(0.01)**_ photocatalyst prepared by facile co-precipitation route. The nanocomposite showed remarkable photocatalytic performance in terms of 2-CP degradation under both visible and direct sunlight in versatile reaction conditions, thus advocating its use as an efficient and robust wastewater treatment candidate.

## Materials and Methods

### Materials

Thiourea (SC(NH_2_)_2_), ferric chloride hexahydrate (FeCl_3_.6H_2_O), ferrous chloride (FeCl_2_), cerium nitrate hexahydrate (Ce(NO_3_)_3_.6H_2_O), potassium carbonate (K_2_CO_3_), sodium hydroxide (NaOH), ethanol (99.9%), ammonium hydroxide (NH_4_OH, 33%), hydrochloric acid (HCl, 37%) obtained from Sigma Aldrich (USA). 2-CP (Sigma Aldrich, USA) was used as pollutant in synthetic water.

### Methods

#### Synthesis of g-C_3_N_4(0.94)_/CeO_2(0.05)_/Fe_3_O_4(0.01)_

g-C_3_N_4_ was done according to the widely used protocol involving direct heating of SC(NH_2_)_2_ at 550 °C for 3 hours^46,47^. CeO_2_ was prepared by the precipitation of cerium nitrate hexahydrate with potassium carbonate solution at 60 °C and at constant pH = 9^48^. The dried powder was calcined at 450 °C up to 3 h with a ramping rate of 5 °C min^−1^. For the preparation of g-C_3_N_4_/CeO_2_ binary composite, different weight percents of CeO_2_ (3%, 5%, 7%) were mixed with g-C_3_N_4_ in ethanol at 100 °C under constant stirring to uniformly distribute CeO_2_ over g-C_3_N_4_ surface. Ethyl alcohol was evaporated and slurry dried at 100 °C to obtain the nanocomposites labelled as GC3, GC5 and GC7. In order to synthesize ternary g-C_3_N_4_/CeO_2_/Fe_3_O_4_ nanocomposite, 1.9 g of GC5 was dissolved in 50 ml of ethanol and water (volume ratio = 1:2) at constant stirring. Then 0.17 mM and 0.087 mM of FeCl_3_.6H_2_O and FeCl_2_ were respectively mixed into the solution at 65 °C and the pH was adjusted at 10 with ammonia solution. Mixture was constantly stirred for another 30 mins (80 °C) and then cooled down at room temperature. Resulting nanocomposite was filtered, washed using ethanol and completely dried in oven at 80 °C^49^. Based on the weight percent of Fe_3_O_4_ i.e. 1%, 3%, 5%, 7% and 10% with respect to GC5, the prepared nanocomposites were labelled as GCF1, GCF3, GCF5, GCF7 and GCF10, respectively.

#### Nanocomposite characterization

Investigation of crystalline nature of as synthesized materials was done using D8 Bruker X-ray Diffractometer varying the incident angle from 20° to 80° using Cu-Kα radiation (λ = 1.5418 nm). XPS measurements were performed in ultra-high vacuum conditions using standard Omicron system equipped with monochromatic Al Kα 1486.7 eV X-ray source operated at 15 KeV at constant analyzer energy of 100 eV for survey scans and 20 eV for detailed scans. Morphology of fabricated photocatalysts was examined by scanning electron microscope (Hitachi S-4800 microscope operated at 20 kV) and JEOL-2100 TEM. The SEM was fitted with EDAX for elemental mapping of the synthesized materials. Raman spectroscopy was performed with a home-made confocal setup fitted with a 532 nm laser. The measurements were performed at 1 mW of excitation power and spectra recorded using an iHR550 imaging spectrometer (from Horiba Scientific). Surface area was calculated through nitrogen physisorption with Nova 2200e (Quantachrome). Diffuse reflectance was recorded in the wavelength ranging from 200 to 800 nm with PerkinElmer, Lambda 750 UV–Vis–NIR spectrophotometer, equipped with integrating sphere. Energy band gap of synthesized photocatalysts were calculated by Kubelka-Munk equation. Room Temperature PL spectra were measured with RF-5301 PC Fluorescence Spectrofluorophotometer (Shimadzu, Japan).

#### Computational study

**Figure 1:**
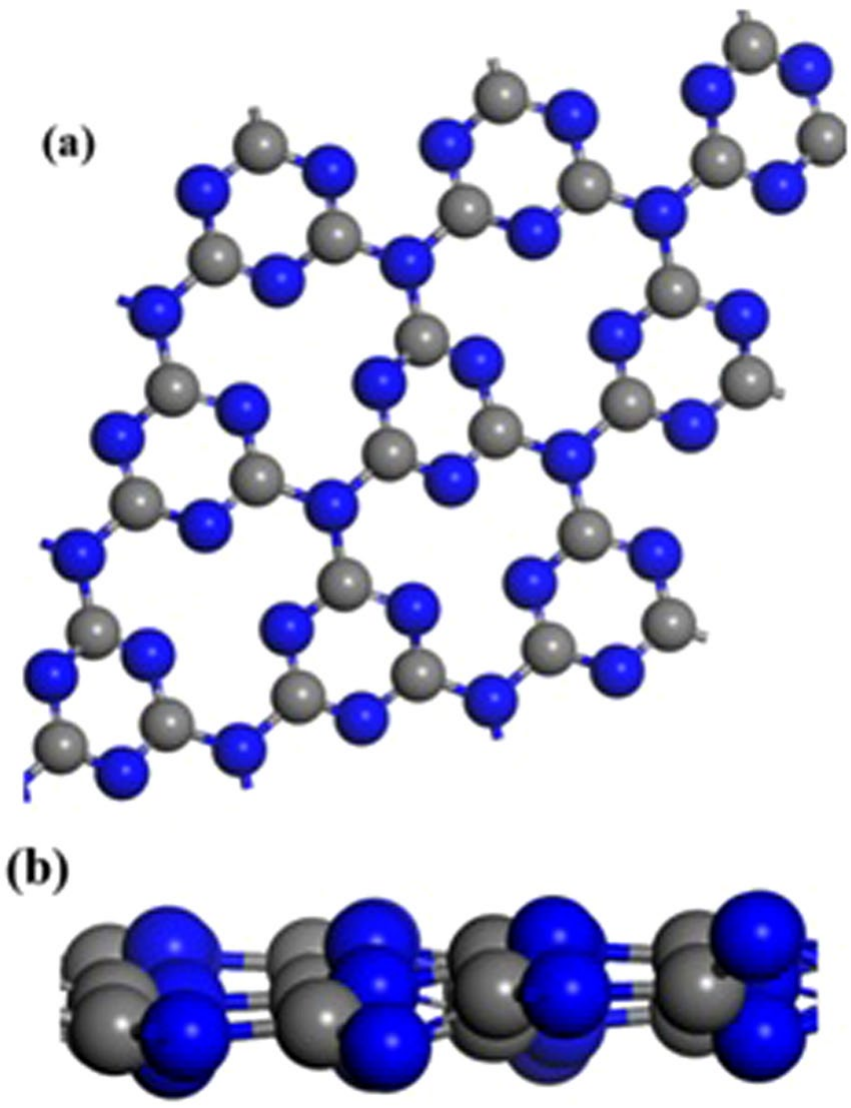
(**a**) Top and (**b**) side view of fully optimized structure of graphitic carbon nitride.

In this study The spin-polarized density functional theory (DFT) was performed using the Vienna ab initio simulation package (VASP)^49–51^. Exchange correlation interaction energy was calculated by using the generalized gradient approximation (GGA) with Perdew-Burke-Ernzerhof (PBE) functional^52^. Projector augmented wave (PAW) pseudopotentials were used to explain the interaction between the valence and core electrons^41^. Valence electrons are described by 4*f* ^1^ 5*d*^1^ 6*s*^2^ for Ce, 3*d*^6^ 6*s*^2^ for Fe, 2*s*^2^ 2*p*^4^ for O, 2*s*^2^ 2*p*^3^ for N and 2*s*^2^ 2*p*^2^ for C. Energy cutoff of 450 eV was employed for treatment of valence electrons. g-C_3_N_4_ was modeled with a super cell consisting of 27 Carbon atoms and 36 Nitrogen atoms. A vacuum space of 15 Å was used to avoid interaction in the complex and its periodic system. The Fully optimized structure of g-C_3_N_4_ is shown in Fig. 1(a,b). For geometry optimizations the Brillion zone integration was calculated with 1 × 1 × 1 k sampling point to gain accuracy. We have used the 5 × 5 × 1 k point sampling for PDOS calculation to gain accuracy for interaction of atomic orbitals near the Fermi Level. All the ions were allowed relaxing till maximum force on any ion is less than 0.02 eV/Å.

#### Photocatalytic experiments

In a typical experiment, 100 ml of 2-CP solution (25 mg L^−1^) was taken into 8 inches diameter Pyrex reaction flasks and catalyst was added in the order of 1 g L^−1^. The suspension was placed in dark for 30 min to equilibrate 2-CP molecules over photocatalyst surface, later the reaction mixture was exposed to direct sun light. During the experiments, reaction vessels were covered with glass covers to ensure only visible light degradation of 2-CP. 5 ml aliquots were sampled after 30 min time intervals and filtered with 0.22 µm syringe filters. Residual concentrations of 2-CP were examined with UV-Vis spectrophotometer at λ = 274 nm. Percentage degradation efficiency (DE %) was determined using Eq. 1:

1 $$DE\, \% =(\frac{{C}_{o}-{C}_{t}}{{C}_{0}})\times 100$$

Here C_o_ = initial pollutant concentration and C_t_ = pollutant concentration at time ‘t’ (min). Influence of different reaction conditions as catalyst dose, Co, pH of solution and reusability studies were also conducted on the selected best photocatalyst. For better insight into the degradation mechanism and to assess the active degrading species separate experiments were designed in lines with the optimal photocatalytic experiments with active species trapping agents. In these experiments t-butanol, p-benzoquinone (BQ) and ethylenediaminetetraacetic acid (EDTA) were used as hydroxyl radical (^•^OH), superoxide radicals (^•^O_2_^−^) and holes (h^+^) scavengers, respectively.

#### Gas chromatography (GC)

For GC analysis of 25 mg.L^−1^ 2-CP, degraded with 1 g.L^−1^ GCF1 under visible light, 5 mL aliquots were taken at specified intervals, filtered through 0.22 μm membrane filters and analyzed for the residual 2-CP concentration using GC. To determine the intermediate products, each test sample (0, 30, 90, 150 min) was extracted thrice using 25 mL of Dichloromethane (DCM). Extract thus obtained was dried using anhydrous Na_2_SO_4_. Samples were subjected to the GC (QP2010 ultra, Shimadzu) having a DB-5ms capillary column using He as carrier. Initial column temperature for 3 min was maintained at 50 °C followed by a gradual temperature increase at 5 °C min^−1^ up to 250 °C. Injector and detector temperatures were fixed at 200 and 260 °C, respectively.

## Results and Discussions

### Structural characterization

**Figure 2:**
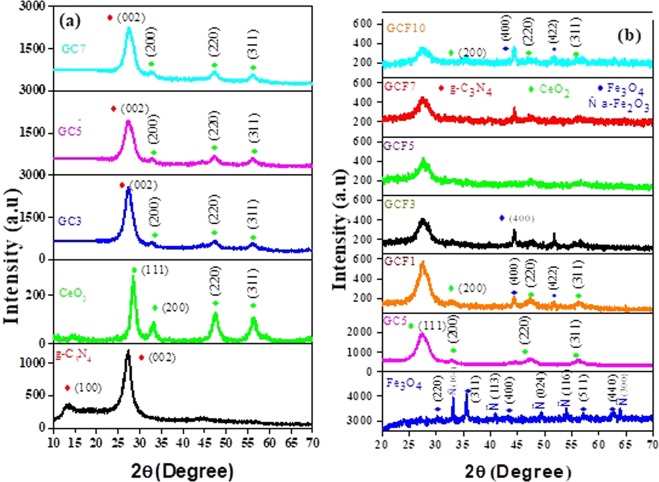
XRD patterns of **(a)** pristine g-C_3_N_4_, CeO_2_ and g-C_3_N_4_/CeO_2_ composites and **(b)** pristine Fe_3_O_4_, g-C_3_N_4_/CeO_2_ (5%) and ternary nanocomposites.

Figure 2a shows the XRD of synthesized components and binary nanocomposites. In pure g-C_3_N_4_, a strong typical peak appears at 27.30° which has an interlayer distance of 0.33 nm is assigned to (002) plane of g-C_3_N_4_, indicating presence of interplaner stacking carbon nitride units^44^. Another diffraction peak with very small intensity at around 13.10° is indexed to (100) and represents tri-s-triazine structure (JCPDS No. 21-1272& 87-1526)^47^. The CeO_2_ diffraction peaks ascribed to the planes of CeO_2_ including main peak (111) and three sister peaks (200), (220) and (311) corresponding to the pure cubic structure^43^ of CeO_2_ (JCPDS No. 043-1002& 34-0394). In case of binary nanocomposites g-C_3_N_4_/CeO_2_ clear indication of sister diffracted planes of CeO_2_ appears (200), (220) and (311). But the main (111) diffracted peak of CeO_2_ at ~28.60° might have been overlapped with the strong plane (002) of g-C_3_N_4_. We noticed the overall intensity of binary nanocomposite g-C_3_N_4_/CeO_2_ has enhanced as we increase the content of CeO_2_ from 3% (GC3), 5% (GC5) and 7% (GC7). Moreover the (100) plane in binary nanocomposite system disappeared which could be the result of CeO_2_ attachment to g-C_3_N_4_ as reported earlier^37^. The XRD patterns of Iron oxide have been presented in Fig. 2(b). The diffracted peaks reflect the mix phase of Fe_3_O_4_ and Fe_2_O_3_ have been observed in pristine iron oxide system. These planes are perfectly corresponding to the cubic spinel Fe_3_O_4_ (JCPDS No. 19-0629, 65-3107, 77-1545 & 3-0863)^46^. But interestingly, in all ternary composites of g-C_3_N_4_/CeO_2_/Fe_3_O_4_ nanosheet samples only the diffracted peaks introduced due to Fe_3_O_4_ phase were noted. The main intense diffracted peak is the combination of (002) from g-C_3_N_4_ and (111) and from CeO_2_ in all samples.

### Morphological and compositional analysis

**Figure 3:**
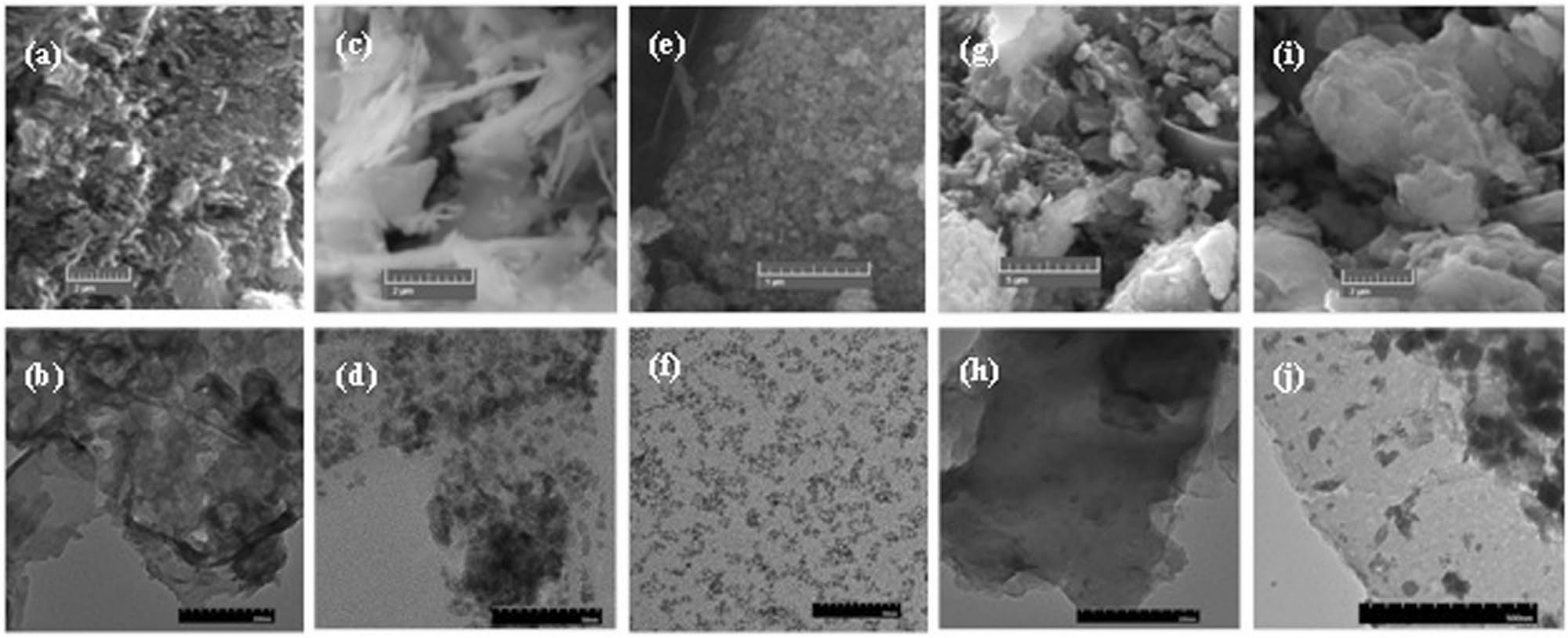
Respective SEM and TEM images (**a,b**) pristine g-C_3_N_4_, (**c**,**d**) CeO_2_, (**e,f**) Fe_3_O_4_, (**g,h**) binary composite g-C_3_N_4_ + 5%CeO_2_ (GC5) and (**i,j**) ternary nanocomposite g-C_3_N_4_ + 5%CeO_2_ + 1%Fe_3_O_4_ (GCF1) photocatalysts.

The morphological properties of the synthesized material were investigating by using SEM and TEM imaging. The SEM images of g-C_3_N_4_, CeO_2_, Fe_3_O_4_, GC5 and GCF1 are displayed in Fig. 3(a,c,e,g,i), respectively. For better elucidation of the particle dimensions and morphology of the synthesized nanocomposites TEM images of g-C_3_N_4_, CeO_2_, Fe_3_O_4_, GC5 and GCF1 are provided in Fig. 3(b,d,f,h,j), respectively. The surface morphology of g-C_3_N_4_ appeared to be composed of a large number of irregular sheets having sufficient small pores which may be due to the discharge volatiles from thiourea decomposition. Such morphology of g-C_3_N_4_ could be due to the aggregation of the sheets of the synthesized samples^47^. The CeO_2_ exhibited very thin flakes like structures with a relatively rough surface while the Fe_3_O_4_ consisted of spheres with the size of ~10–20 nm as reported in similar studies^49,53^. In case of the nanocomposites, the TEM images clearly indicate that the sheets of g-C_3_N_4_ covered with the CeO_2_ flakes and Fe_3_O_4_ nanoparticles.

**Figure 4:**
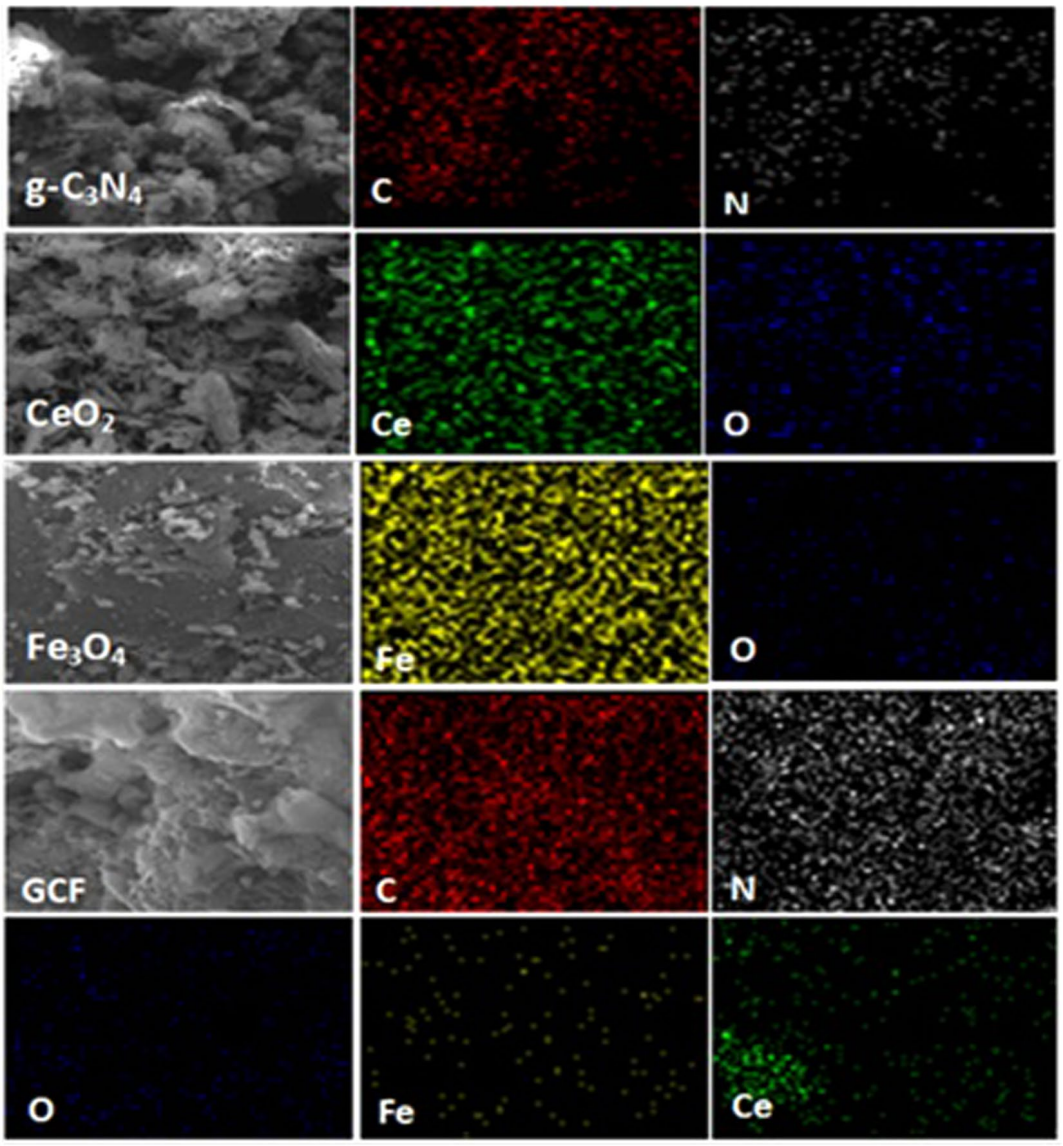
Elemental mapping of prepared pristine and nanocomposite photocatalysts.

The elemental composition and distribution in the prepared samples were also investigated to confirm the morphology of the synthesized materials. Elemental mapping of the samples is presented in Fig. 4. The high purity of g-C_3_N_4_, CeO_2_ and Fe_3_O_4_ nanoparticles was confirmed in the respective samples. Similar results were found in the case of GC5 and GCF1 which not only displayed the high purity but also showed the homogeneous distribution of elements within the composites^54–56^. The surface area determinations of the nanocomposite photocatalysts exhibited a higher surface area for bulk g-C_3_N_4_ i.e., 17.421 m^2^.g^−1^ ^55^, CeO_2_ and 5% CeO_2_ exhibited surface area of 30.229 and 29.444 m².g^−1^, respectively confirming the incorporation of CeO_2_ within the g-C_3_N_4_ matrix.

**Figure 5:**
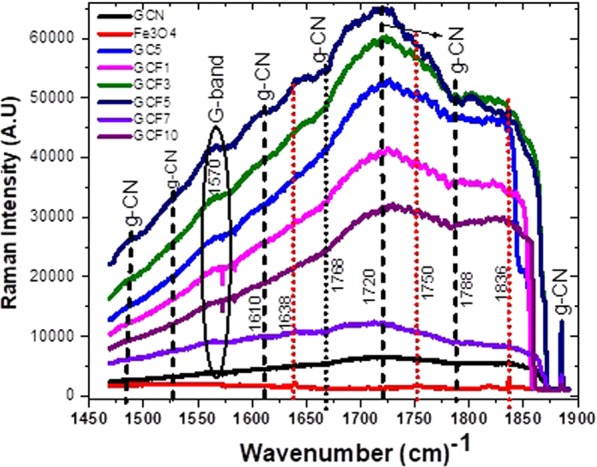
Resolved Raman Spectra of –gC_3_N_4_ nanosheets composite series.

The amplified Raman spectra of the synthesized nanocomposite series is provided in Fig. 5. The peak located 1485 cm^−1^ arises from the vibration modes of CN clearly visible in all the composite samples including GC and GCF (1–10), signifying that there was no phase change during the composite formation might be due to CeO_2_ and Fe_3_O_4_ which is in complete agreement with the XRD results. A series of modes could be detected in the amplified Raman spectrum of GCF1 including the D and G band located around 1405 cm^−1^ and 1570 cm^−1^ respectively. The peaks corresponding to the CH_3_ bending (scissor deformation, 1449 cm^−1^) from amide 2 N–H deformation (1544 cm^−1^), and amide 1 C=O stretching (1646 cm^−1^)^57^ are characteristic of g-C_3_N_4_.

### Chemical characterization

**Figure 6:**
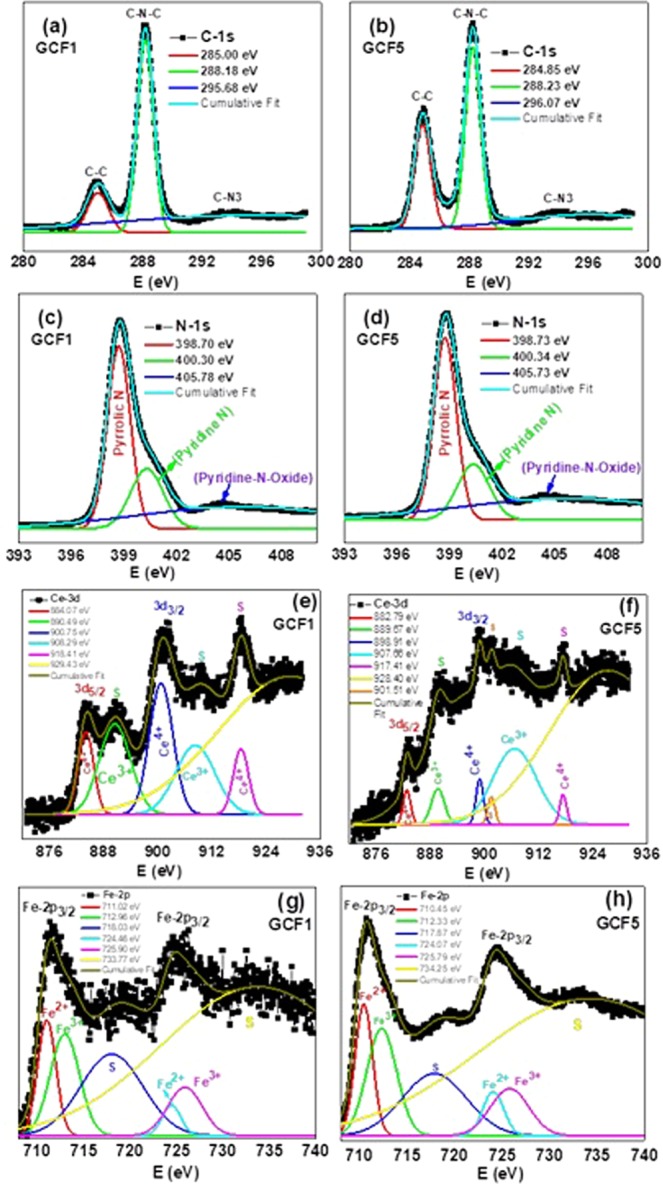
High resolution XPS spectra of GCF1vs GCF5 **(a**,**b)** C-1s, (**c**,**d**) N-1s, (**e**,**f**) Ce-3d, and (**g**,**h**) Fe-2p peaks.

High resolution (HR) XPS investigations identified the oxidation states of complete series of samples. To understand the photocatalytic activity, here we have presented the XPS data of GCF1 and GCF5 samples. The atomic percentage of C (38.92%), O (7.69%), Ce (0.76%), N (50.69%), Fe (1.94%) were obtained for GCF1 sample from XPS measurement. Similarly the measured atomic parentage values of C (35.57%), O (19.72%), Ce (1.09%), N (34.79%), Fe (8.84%) were obtained for GCF5 sample. The presence of higher oxygen content may be due to the surface oxidation by adsorption of environmental oxygen species on the surface of samples. The Fig. 6(a,b) presented C-1s HR-XPS for GCF1 and GCF5 composites nanosheets, respectively. We found a higher intensity peak located at 288.18 eV corresponds to sp^2^-bonded carbon (C-N-C) in GCF1 sample as compared to GCF5, while observed an opposite trend of intensities of the peak centered at 284.85 eV that might be accredited to C=C synchronization^54^. Additionally, a minor peak at 295.68 eV associated with CN_3_ has been detected having almost same intensity^55^. The high resolution XPS peak of N-1s for GCF1 and GCF5 samples has been presented in Fig. 6(c,d), respectively. The central peak position formulated at ≈398.70 eV that correspond to C-N-C geometry. An unconventional insignificant intense peak at location 405.78 eV matching to Pyridine-N-oxide has been perceived in both ternary composite samples^56^. We noticed that the intensity of GCF5 is lower as compared to GCF1 for N-1s core spectra. These observations may predict that the higher intensities of N-1s and C-1s for GCF1 as compared to GCF5 will possible play a role to enhance the photolytic activity. The Ce-3d XPS spectrum was measured in order to approximate quantification for the comparative abundances of Ce^4+^ and Ce^3+^ species. Figure 6(e,f) revealed XPS core spectra for the ternary nanosheets samples GCF1 and GCF5, respectively. We noticed peaks that can be divided easily in the 3d_3*/*2_ and 3d_5*/*2_ spectroscopic terms along with two satellite peaks of Ce^4+^ species and the other two satellites peaks may be originated due to Ce^3+^ ions. These observations are in good agreement with previous reports. HR-XPS measurements were performed to verify Fe 2p core level photoemission spectrum of ions in the GCF1 and GFC5 nanosheets as illustrated in Fig. 6(g,h), respectively. The reported value of metallic Fe has a peak position of Fe 2p_1/2_ at 719.9 eV and Fe 2p_3/2_ at 706.5 eV; FeO at 722.3 eV and 709.3 eV, and Fe_2_O_3_ at 724.9 eV and 710.5 eV, respectively.

### Diffuse reflectance and photoluminescence studies

**Figure 7:**
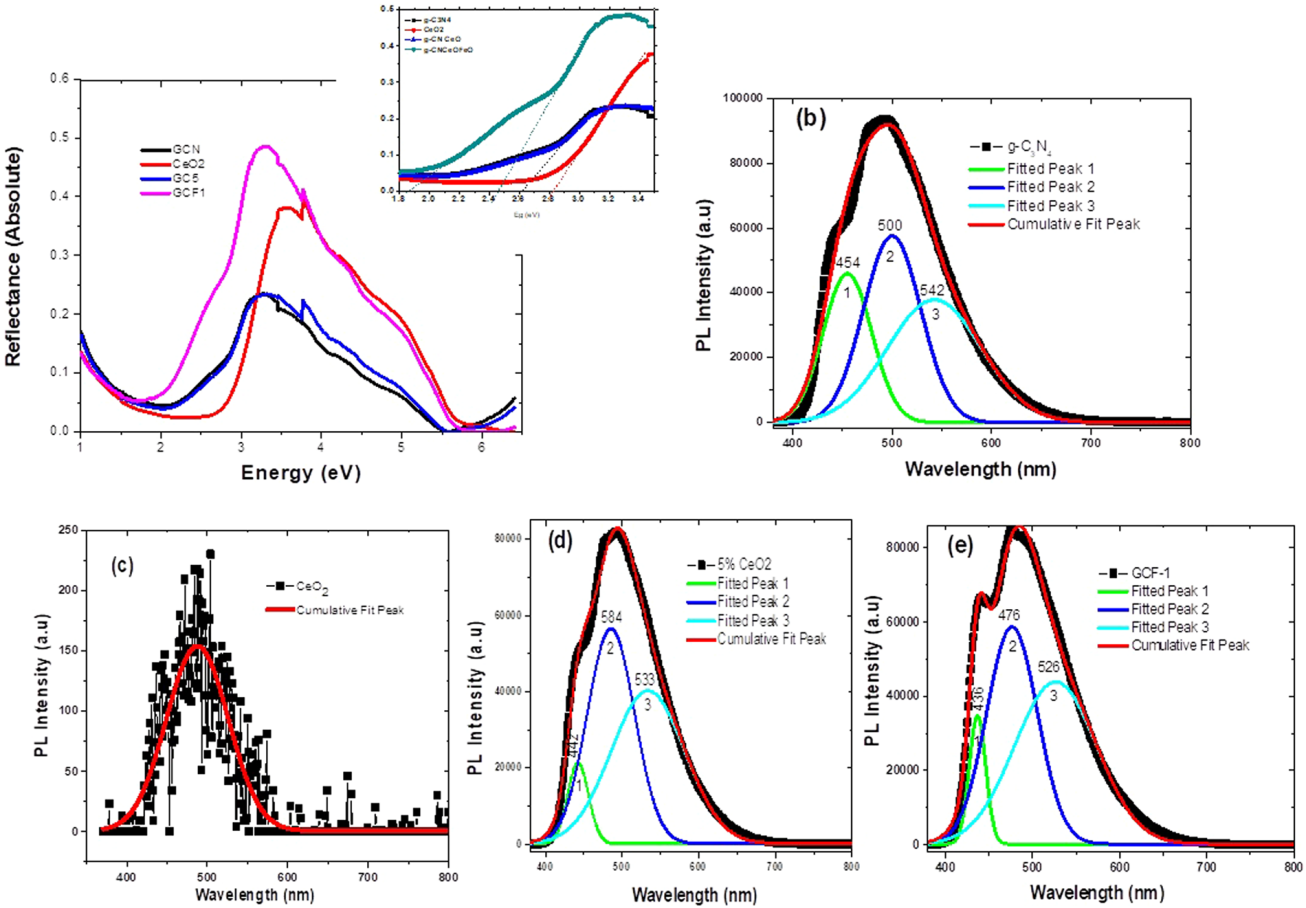
(**a**) Diffuse reflectance spectra and (inset) band gap energy calculated for GCN, CeO_2_, GC5 and GCF1; Room temperature photoluminescence spectra of: (**b)** pristine g-C_3_N_4_ nanosheets; (**c)** CeO_2_ nanosheets; (**d)** binary composite g-C_3_N_4_/CeO_2_ nanosheets. (**e)** Ternary composites of g-C_3_N_4_/CeO_2_/Fe_3_O_4_ nanosheets.

Figure 7(a) shows the DRS of g-C_3_N_4_, CeO_2_, GC5 and GCF1. For band gap calculation (*F*(*R*) × *hν*)^2^ and (*F*(*R*) × *h)*^1/2^ vs. *hν* were plotted the indirect energy band gaps of g-C_3_N_4_, CeO_2_, GC5 and GCF1. From the plot the band gap energy (*E*_*g*_) was determined from the linear region of the plot on *x*-axis. The inset shows the reflectance band edges corresponding to band gap energies of 2.68 eV, 2.82 eV, 2.62 eV and 2.45 eV for g-C_3_N_4_, CeO_2_, GC5 and GCF1, respectively. The second band edge in GCF1 corresponding to E_g_ 1.84 eV may represent Fe_3_O_4_ as reported in literature^58^. These results suggest that GCF1 is an indirect band gap semiconductor. The RT-PL spectra (@ λ = 325 nm) for the pristine g-C_3_N_4_, CeO_2_, binary GC5 and ternary GCF1 nanosheet samples are presented in Fig. 7(b–e), respectively. We noticed that bare g-C_3_N_4_ and hybrid nanosheets exhibit intense emission PL spectra as compared to pristine CeO_2_ nanosheets. The diminishing in emission peak strength for binary and ternary nanocomposites is due to restrained e^−^/h^+^ recombination within the g-C_3_N_4_/CeO_2_ and g-C_3_N_4_/CeO_2_/Fe_3_O_4_ heterojunctions, which further indicate a successful charge separation. The FWHM of binary nanocomposite samples was less than pure g-C_3_N_4_ while the ternary nanocomposite samples the case was reverse. The variation in the values of FWHM may be due to the variable defects concentration in each sample. Consequently, the data was deconvoluted into three fitted peaks for bare, binary and ternary nanosheets samples. These fitted peaks with varied peak positions were assigned names peak-1, peak-2 and peak-3 as illustrated in Fig. 7(b,d,e). Gaussian fitting of PL emission bands reflects the different type of possible defects in each sample. Figure 7(b) evidenced for the line profile investigation of the g-C_3_N_4_ sample, which includes the emission center peak-1 (454 nm, 2.73 eV), peak-2 (500 nm, 2.48 eV) and peak-3 (542 nm, 2.28 eV). Similarly, the central emission of peak-1 for binary sample (442 nm, 2.80 eV) and ternary sample (436 nm, 2.84 eV) indicates clearer variation in band gap.

### DFT- geometry and electronic structure of binary and ternary nanocomposites

#### Geometry and electronic structure of Ce- g/C_3_N_4_monolayer

**Figure 8:**
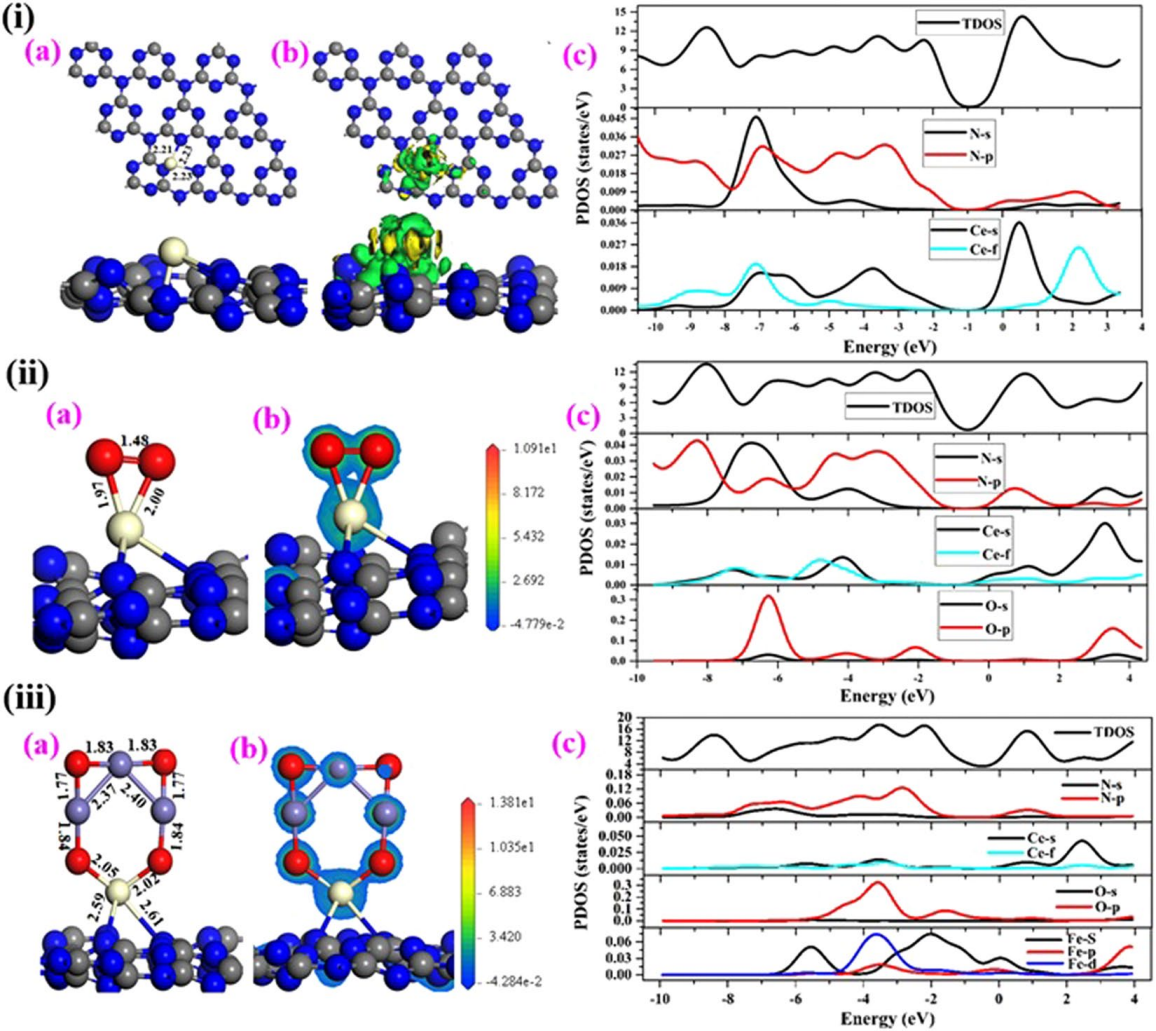
(i) Single Ce atom, (ii) CeO_2_ and (iii) Fe_3_O_4_ doped on graphitic carbon nitride (g/C_3_N_4_) monolayer. (**a**) Optimized geometry. (**b**) Charge density differences, for the contour plots, the charge accumulation regions are rendered in green. The contour value of the charge difference density is ±0.05 a.u. and (**c**) The spin-polarized partial density of states (PDOS) projected on TDOS (black), C-2*s* (black) and C-2*p* (red), N-2*s* (black) and N-2*p* (red)*, Ce-*6*s* (black) and Ce-4*f* (cyan), O-*2s* (black) and O-2*p* (red) and Fe-4*s* (black), Fe-4*p* (red) and Fe-3*d* (blue) states. The Fermi level is set to zero.

The optimized geometry of Ce- g-C_3_N_4_ monolayer shown in the Fig. 8i(a). It is found that the Ce atom preferred to locate on Hollow site of g-C_3_N_4_ monolayer is bonded with three Nitrogen atom with the bond distance of Fe-N (2.23 Å) and (2.21 Å), respectively. The Fig. 8i(b) shows the charge density differences of Ce-g-C_3_N_4_ monolayer, which shows the significant charge density accumulation and depletion region between the Ce atom and its neighboring nitrogen atoms. To clearly understand the electronic structure, Fig. 8i(c) demonstrates the partial density of states (PDOS) of Ce-g-C_3_N_4_) monolayer. Figure 8i(a) also depicts that strong interaction between Ce and N atoms. Further confirmed by overlapping peaks of Ce-4*f*, 6*s* and N-2*p* orbitals near to the fermi level suggests, higher reactivity of Ce- g/C_3_N_4_ monolayer.

#### Geometry and electronic structure of CeO_2_ doped on g/C_3_N_4_ monolayer

Figure 8ii(a) illustrates the most energetically preferred adsorption complex of CeO_2_ on g-C_3_N_4_ monolayer. The bond distance between the Ce-O is 2.00 Å and 1.97 Å respectively. The bond distance of O-O is slightly elongated from 1.23 (Free O_2_) to 1.48 Å due to the charge transfer from Ce to O_2_ and activate the O_2_ molecule. The Fig. 8ii(b) shows the charge density differences of CeO_2_ doped on graphitic carbon nitride (g/C_3_N_4_) monolayer. The charge density transfers occur from the Ce 4f, 6*s* orbitals to 2π^*^ antibonding orbitals of O. The Fig. 8ii(c) displays the PDOS curves of the CeO_2_ adsorption on the g-C_3_N_4_ monolayer. The strong mixing observed between 4*f* and 6*s* orbitals of Ce and 2π^*^ orbitals of O and N near to the fermi level can be clearly seen, which is the significant wreaking of the O-O bond distance and strong binding of CeO_2_ with g-C_3_N_4_.

#### Geometry and electronic structure of Fe_3_O_4_ doped on Ce- g-C_3_N_4_ monolayer

The most stable adsorption configuration of Fe_3_O_4_ doped on Ce-g-C_3_N_4_ monolayer shown in Fig. 8iii(a). The observed Ce-O bond length is (2.02 Å and 2.02 Å) and the O-Fe is (1.83 Å, 1.83 Å and 1.62 Å) respectively. The Fig. 8iii(b) shows the charge density differences of Fe_3_O_4_ doped on Ce-graphitic carbon nitride (g/C_3_N_4_) monolayer. The charge density transfers occur from the Ce 4f, 6*s* orbitals to 2π^*^ antibonding orbitals of O. Figure shows the charge density accumulation and depletion region between the Ce atom and its neighboring oxygen atoms. The Fig. 8iii(c) shows the partial density of state (PDOS) curves of the Fe_3_O_4_ doped on Ce- g-C_3_N_4_ monolayer. The strong mixing observed between the 3*d* orbitals of Fe, 4*f* and 6*s* orbitals of Ce and 2π^*^ orbitals of O near to the fermi level can be clearly seen. Furthermore, the strong interaction between the Fe, Ce and O atoms are confirmed by the overlapping peaks near to the fermi level. The fermi level is set to be zero.

### Photocatalysis of 2-CP

**Figure 9:**
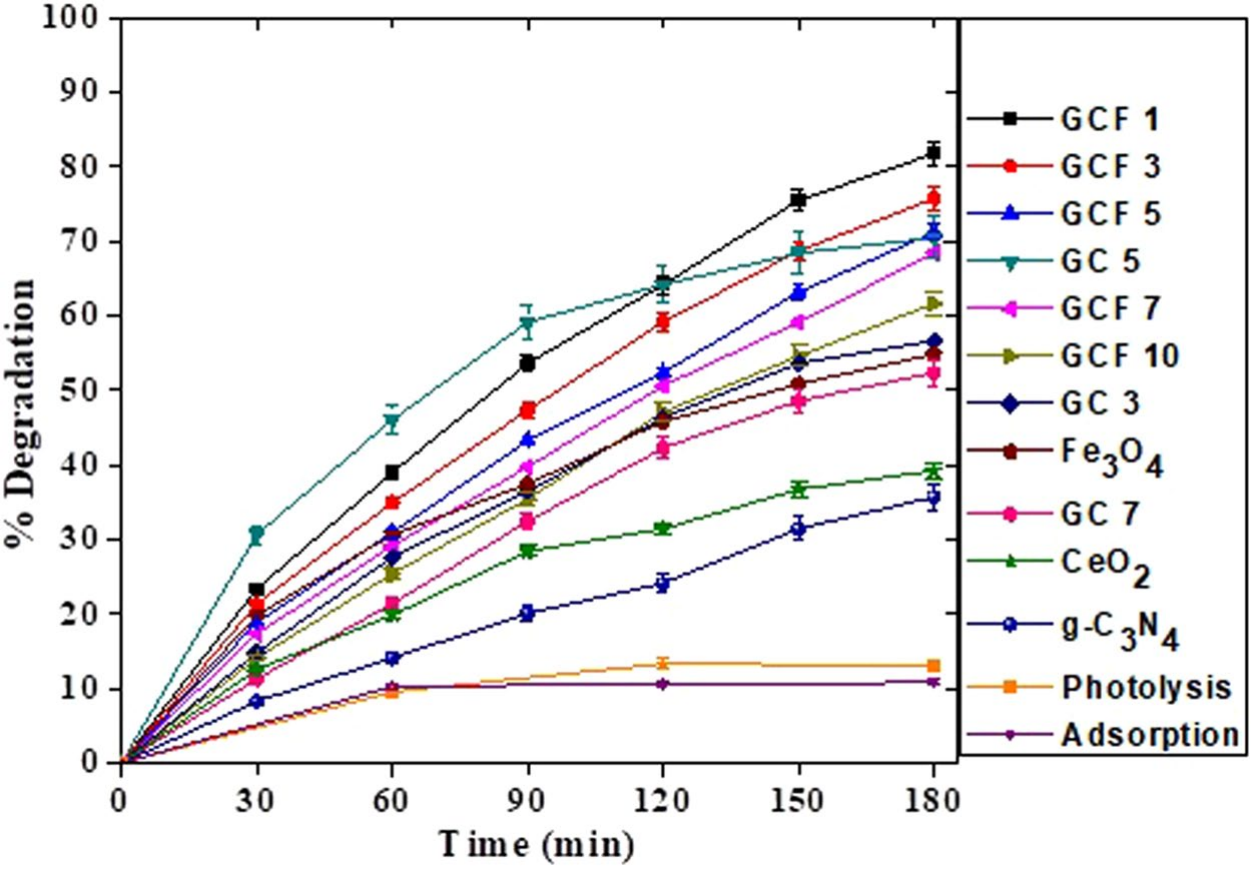
Photocatalytic 2-CP degradation using pristine and modified nanomaterials.

The photocatalytic degradation of 2-CP under direct sunlight using pristine and modified binary and ternary nanocomposite photocatalysts was investigated as shown in Fig. 9. The photocatalytic experiments evidenced rapid increase in the degradation of 2-CP by using 5% g-C_3_N_4_/CeO_2_ (GC5) as compare to other pristine components and binary nanocomposites. Further increase in the CeO_2_ content up to 7%, manifested lowering the photocatalytic degradation efficiency signifying light absorption hindrance effect due to excessive CeO_2_ content. Excessive CeO_2_ content was also harmful for the efficient electron hole separation and decreased the active sites on the nanocomposite^35^. Therefore, further catalyst modifications with Fe_3_O_4_ were carried upon GC5.

Figure 9 also showed that among all the ternary nanocomposites, highest degradation of 2-CP was achieved upon using g-C_3_N_4(0.94)_/CeO_2(0.05)_/Fe_3_O_4(0.01**)**_ i.e., GCF1 and the degradation efficiency decreased as the Fe_3_O_4_ percentage increased from 1–10%. Increased amount of Fe_3_O_4_ might have acted as a recombination centre for the photo-generated e^−^/h^+^ which ultimately decreased the photocatalytic efficiency of the nanocomposites^36^. Enhanced photocatalysis of pristine g-C_3_N_4_ by modifying its surface with CeO_2_ and Fe_3_O_4_ can be explained firstly as the addition of CeO_2_ and Fe_3_O_4_ leads to modify the colour of this material, leading to the improved harvesting of visible light region as shown in UV-Vis DRS. Secondly, formation of semiconductor-semiconductor heterojunction of g-C_3_N_4_ with other semiconductors components resulted in effective electron hole separation in the nanocomposite and increased generation of oxidant species for the degradation of 2-CP^35^.

**Figure 10:**
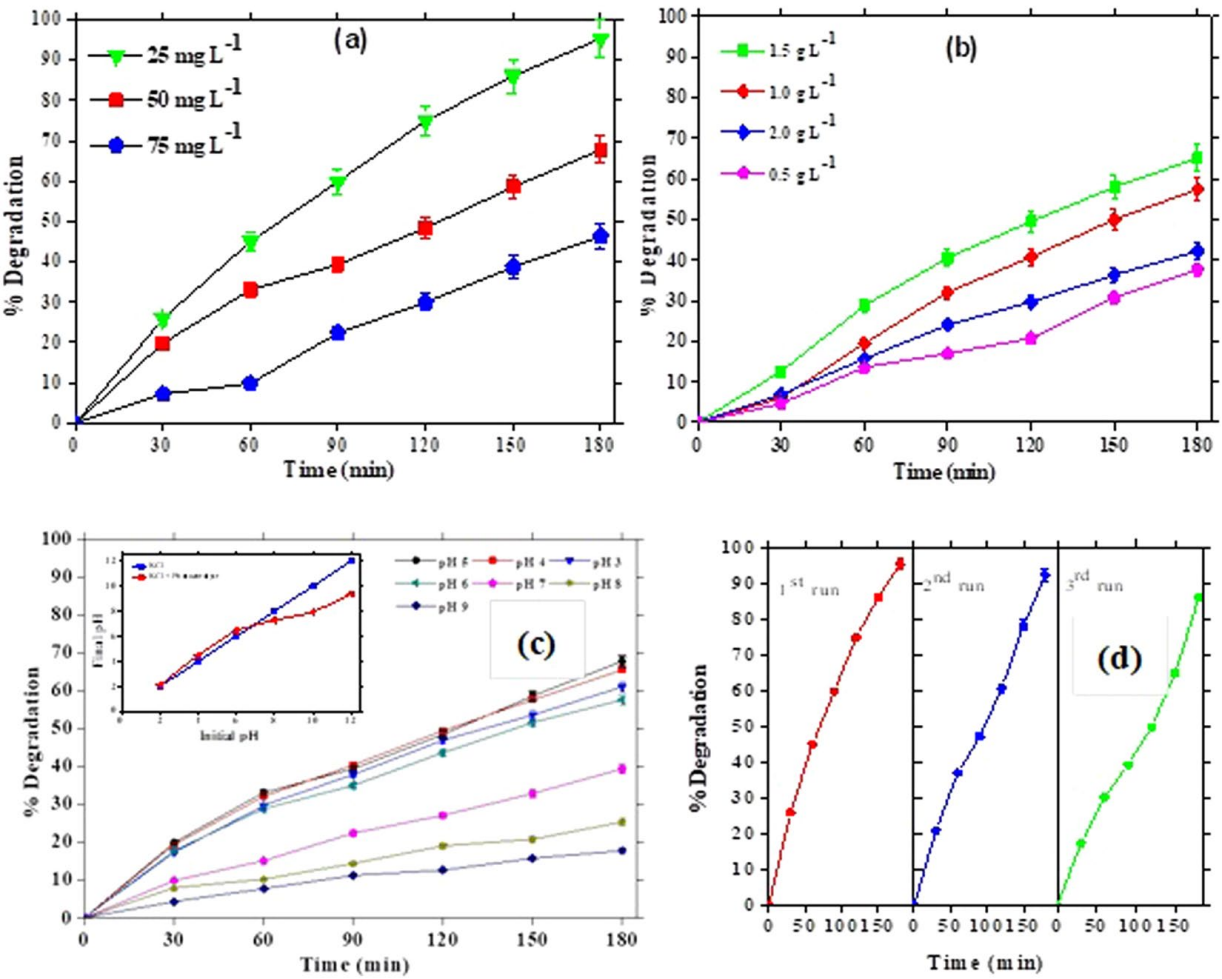
Photocatalytic activity influenced by; (**a)** initial 2-CP concentration; (**b)** catalyst dosage**; (c)** variable solution pH; (**d)** catalysts reusability.

Figure 10(a) shows the results in terms of 2-CP degradation as a function of irradiation time. Degradation efficiency showed considerable decline with increasing 2-CP concentration up to 75 mg L^−1^. Highest degradation at 25 mg L^−1^ was achieved due to availability of higher surface area per unit 2-CP molecules at lower pollutant concentration. Upon increase in 2-CP concentration the number of pollutant molecules increased while the number of catalyst active sits for pollutant attachment remained constant thus decreasing the overall degradation efficiency^59^. Increase in pollutant concentration not only decreased the surface area of photocatalyst but also restrained the light utilization by the photocatalyst for the generation of reactive species like hydroxyl radicals^14^. Catalyst dose is one of the most important factors which significantly affects the degradation efficiency of photocatalytic process. A series of experiments were conducted by using varied amounts of GCF1 (from 0.5–2 g.L^−1^) over constant 2-CP concentration of 50 mg L^−1^. From Fig. 10(b), it is evident that enhanced photocatalytic activity was achieved with increase in catalyst dose from 0.5–1.5 g.L^−1^ as increased catalyst dose enhances the number of active site which results in generation of more reactive red-ox species^58^. But as we move from 1.5–2 g.L^−1^ catalyst dose, the degradation efficiency clearly decreased owing to the light screening effect of the additional catalyst dose which reduces the surface area of photocatalyst for light illumination^37^, in turn reducing the degradation efficiency of the photocatalyst. Figure 10(c) illustrates reduction in GCF1 photocatalysis of 50 mg.L^−1^ 2-CP with increase in pH. The degradation rate at pH 3 and 4 was low due to the competition between the 2-CP molecules and excess Cl^−^ ions (from HCl used to adjust the pH). On the contrary, lower degradation rate at basic conditions could be the result of electrostatic repulsion between the negatively charge GCF1 (pHzpc = 6.9) and phenolate ions. This decreases the adsorption of 2-CP molecules on the surface of the catalyst and negatively affects the degradation rate^35,59^. To evaluate stability of nanocomposites additional runs of 2-CP degradation (25 mg.L^−1^) were conducted at optimum conditions. Figure 10(d) illustrates the degradation efficiency of reused catalyst in three successive runs. The photocatalytic degradation efficiency of GCF1 declined ordinarily after the second and third reuse of the photocatalyst with only 8% reduction in the photocatalytic efficiency. However, only 8% loss in activity after three time use and in absence of any regeneration procedure is a testament of catalyst stability and retention of high catalytic activity.

Photocatalytic degradation of 2-CP is evaluated with first order, second order and zero order reaction kinetics^60,61^. Basic relationships of these equations are given below in respective order:

2 $$ln(\frac{{C}_{^\circ }}{C})=kt$$

3 $$\frac{1}{C}=\frac{1}{{C}_{o}}+kt$$

4 $${C}_{o}-C=kt$$

Here, C_o_ = 2-CP (initial concentration), C = 2-CP (residual concentration after solar exposure time ‘t’ (min)) while k is rate constant (min^−1^) of respective equations.

Kinetic plot for first order ‘(ln (C_o_/C)’ vs time), second order equation (1/C vs time) and zero order equations (C_o_-C vs time) at variable reaction parameters demonstrated that the ternary nanocomposite followed pseudo first-order reaction kinetics with 2.55 and 4.04 times increased rate of reactions compared to pristine Fe_3_O_4_ and CeO_2_, with highest rate constant value of 18.2 × 10^−3^ min^−1^ as shown in Table 1.

**Table 1:** Kinetic constant values explaining the effect of catalyst dose, pH and 2-CP concentration over ternary nanocomposite GCF1.

Experiment	Conditions	First order		Second order		Zero order	
		k (min^−1^)	R^2^	k (min^−1^)	R^2^	k(min^−1^)	R^2^
^a^Effect of 2-CP conc. (mg L^−1^)	25	**0.0182**	0.9726	0.0047	0.7527	0.1173	0.9613
	50	0.0060	0.9991	0.0002	0.9838	0.1711	0.9765
	75	0.0038	0.9813	0.00007	0.9654	0.2068	0.9856
^b^Effect of pH	5	**0.0059**	0.9799	0.0002	0.9305	0.1549	0.9940
	4	0.0055	0.9956	0.0002	0.9656	0.1510	0.9930
	3	0.0049	0.9983	0.0002	0.9816	0.1427	0.9893
	6	0.0044	0.9962	0.0002	0.9807	0.1316	0.9933
	7	0.0026	0.994	0.00007	0.9831	0.0979	0.9977
	8	0.0014	0.9871	0.00003	0.9837	0.0586	0.9887
	9	0.001	0.9897	0.00002	0.992	0.0442	0.9865
^c^Effect of catalyst conc. (g L^−1^)	1.5	0.006	0.9991	0.0002	0.9838	0.1711	0.9765
	1.0	0.0053	0.9994	0.0002	0.9846	0.1703	0.9873
	2.0	0.0031	0.9989	0.00009	0.9948	0.1163	0.9927
	0.5	0.0027	0.964	0.00007	0.9449	0.1052	0.9755

^a^(Catalyst dosage: 1 g L^−1^, Solution pH = 5).

^b^(2-CP initial concentration: 50 mg L^−1^; Catalyst dosage: 1 g L^−1^).

^c^(2-CP = 50 mg L^−1^, pH = 5).

**Figure 11:**
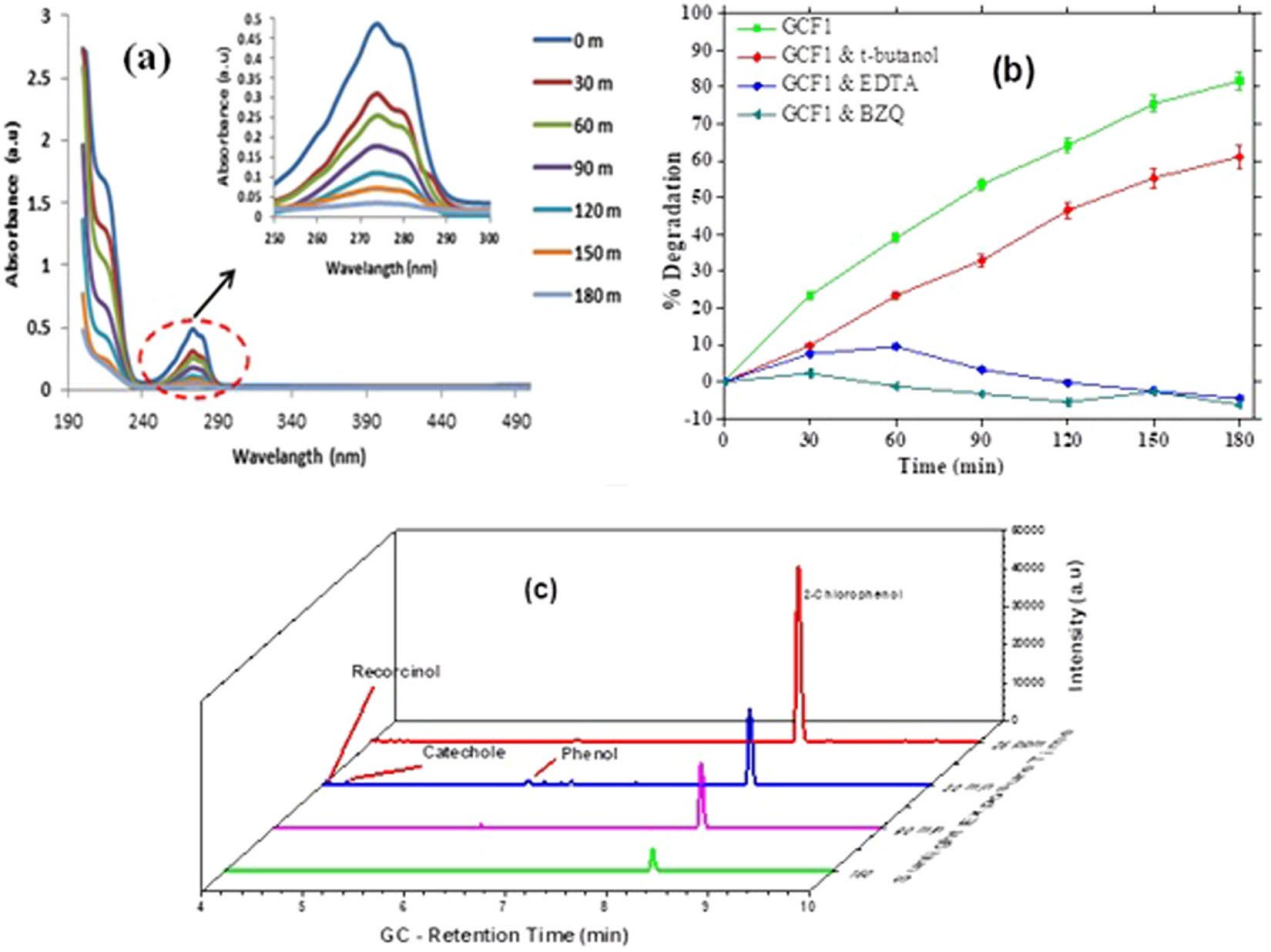
**(a**) UV-Visible absorption spectra of 2-CP degradation over GCF1. **(b)** Influence of reactive species scavenging on photocatalytic activity; **(c)** GC Analysis results.

Although the degradation process is clearly illustrated form the complete scan spectra of degradation samples of the photocatalytic process (Fig. 11(a)); to determine the photocatalytic mechanism of 2-CP degradation over GCF1 BZQ, tert-butyl alcohol and Na_2_-EDTA were used as OH^•^, O_2_^•−^ and h^+^ scavengers, respectively^62^. As shown in Fig. 11(b), upon using Na_2_-EDTA a remarkable decrease on the degradation efficiency of 2-CP was observed. In these experiments, only up to 9% 2-CP was degraded in initial 60 min. The addition of BZQ into the photocatalytic experiment also clearly showed inhibitory influence towards the degradation to <1%, whereas the presence of tert-butyl alcohol had a comparatively lower but observable inhibitory effect on overall photocatalysis. The CB potential of g-C_3_N_4_ is more negative than potential of O_2_/^•^O_2_^−^ (−0.33 eV). Therefore, adsorbed oxygen over g-C_3_N_4_ was reduced to ^•^O_2_^−^ by capturing electron. The potential for O_2_/H_2_O_2_ (+0.695 eV) is higher than the CB energy of g-C_3_N_4_ and CeO_2_. Consequently, the electrons in CB of g-C_3_N_4_ and CeO_2_ react with adsorbed oxygen to produce hydrogen per-oxide. The produced hydrogen peroxide molecules generate ^•^OH radicals by capturing electrons in another step. However, oxidation of hydroxide ions (E°_−OH/OH_° = +2.38 eV) and molecules of water (E^◦^_H2O/OH_^◦^ = +2.72 eV) to hydroxyl radicals do not take place on the VB of g-C_3_N_4_ and CeO_2_ ^37^. Observable decline was found in the given order; O_2_^•−^ > h^+^ > OH^•^. The band gap (Eg) of g-C_3_N_4_, CeO_2_ and Fe_3_O_4_ were ~2.7 eV, ~2.92 eV and ~1.5 eV, respectively which results in the excitation upon exposure to visible light irradiation to generate the electrons and holes^37^. The valance band and conduction band of g-C_3_N_4_ have more negative potential compared to the CeO_2_ and Fe_3_O_4_ due to which the photo generated electrons produced on CB of g-C_3_N_4_ transfer towards the CB of CeO_2_ and subsequently towards the CB of Fe_3_O_4_. Similarly the holes generated in the VB of CeO_2_ transfer to the VB of g-C_3_N_4_. Consequently, the electron-hole recombination process is minimized due to effective charge separation. Furthermore the photogenerated electrons in the CB of Fe_3_O_4_ can react with the ubiquitous molecular oxygen to form the superoxide radical O_2_^•−^, which can contribute to the decomposition of 2-CP while the photo generated h^+^ oxidize H_2_O and OH^−^ ion into free •OH radicals^41,42^. Finally, 2-CP molecules are degraded by the holes, superoxide radicals and hydroxyl radicals.

The gas chromatographic analysis of optimized photocatalytic study complementing our findings are provided in Fig. 11(c). The degradation of 2-CP can be clearly visualized through decline in the GC peak intensity at retention time 8.31 min. From the results it can be seen that phenol, catechol and hydroquinone were formed as intermediate products during the degradation process by hydroxylation and de-halogenations mechanisms proposed in similar studies^63–65^. However at later stages of the photocatalytic process (at time 150 min) the intermediate species disappear indicating their mineralization along with 2-CP and complete mineralization at 180 min.

## Conclusions

Novel g-C_3_N_4_ based magnetically separable and visible light active ternary composite nanosheets for photocatalysis of 2-CP polluted water is reported. TEM imaging showed that the GCF nanocomposite exhibited high purity and crystallinity with uniform dispersion of CeO_2_ and Fe_3_O_4_ nanoparticles over g-C_3_N_4_ nanosheets. The DFT predicted charge density transfers occur from the Ce 4f, 5*s* orbitals to 2π^*^ antibonding orbitals of O and N. The PDOS curves of Fe_3_O_4_ doped on g-C_3_N_4_/CeO_2_ monolayer suggested strong interaction between Fe, Ce and O atoms confirmed by the overlapping peaks near to the fermi level which favor the photocatalytic reactions over the nanocomposite. The XRD and XPS determinations supported the existence of Fe_3_O_4_ in the composite as the dominant crystalline structure and the obtained GCF1 nanocomposite showed excellent visible light photocatalytic activity towards 2-CP breakdown at concentrations from 25–75 mg.L^−1^. Complete mineralization was observed within 180 min of sun light exposure with highest rate constant value of 18.2 × 10^−3^ min^−1^. The catalyst showed high stability over extreme pH conditions and repeatability test showed that GCF1 retained 92.5% activity after three times reuse confirming the robustness of the photocatalysts system. In comparison, pristine g-C_3_N_4_, CeO_2_, Fe_3_O_4_ and all other binary and ternary nanocomposites synthesized, best photocatalytic performance was obtained by using GCF1. The GCF1 catalyst also exhibited very swift reaction rate with rate constant value of 60 × 10^−4^ min^−1^ and regression co-efficient value of 0.9991 at pH 5. This study describes an easy fabrication of novel ternary composite and provides an innovative solution for the treatment of 2-CP contaminated wastewater with an additional advantage of easy recovery and reusability simply through applying a weak external magnetic field.
